# Immunological, Viral, Environmental, and Individual Factors Modulating Lung Immune Response to Respiratory Syncytial Virus

**DOI:** 10.1155/2015/875723

**Published:** 2015-05-06

**Authors:** Silvia Vandini, Paolo Bottau, Giacomo Faldella, Marcello Lanari

**Affiliations:** ^1^Neonatology and Neonatal Intensive Care Unit, S. Orsola-Malpighi Hospital, University of Bologna, Via Massarenti 11, 40138 Bologna, Italy; ^2^Pediatrics and Neonatology Unit, Imola Hospital, Via Montericco 4, 40026 Imola, Italy

## Abstract

Respiratory syncytial virus is a worldwide pathogen agent responsible for frequent respiratory tract infections that may become severe and potentially lethal in high risk infants and adults. Several studies have been performed to investigate the immune response that determines the clinical course of the infection. In the present paper, we review the literature on viral, environmental, and host factors influencing virus response; the mechanisms of the immune response; and the action of nonimmunological factors. These mechanisms have often been studied in animal models and in the present review we also summarize the main findings obtained from animal models as well as the limits of each of these models. Understanding the lung response involved in the pathogenesis of these respiratory infections could be useful in improving the preventive strategies against respiratory syncytial virus.

## 1. Introduction

Respiratory syncytial virus (RSV) is the most important cause of bronchiolitis and severe acute respiratory infections in infants younger than 2 years [1, 2]; mortality rate for RSV bronchiolitis is nine-fold higher than mortality due to influenza virus during the first year of life [3]. The worldwide burden of acute lower respiratory tract infections (LRTI) due to RSV was estimated to be at least 33.8 million in children <5 years of age in 2005 (22% of all episodes of LRTI in young children). Moreover, the estimated hospitalization rate was 3.4 million/year and the estimated mortality was 66,000–199,000 (99% of deaths occurred in developing countries) [2]. RSV is a highly contagious virus and is easily spread in hospitals, homes, and nurseries, despite being less cytopathic and less invasive than the influenza A virus [4]. In addition, RSV infections have a heavy impact on healthcare resources: Paramore et al. [5] reported approximately 86,000 hospitalisation cases, 402,000 emergency room visits, 1.7 million office visits, and 236,000 hospital outpatient visits in children <5 years during 2000, with estimated costs totalling almost $652 million USD.

Patients at high risk of severe RSV infections include infants younger than one year, immunocompromised patients, elderly people (≥65 years old), and high-risk adults (those with chronic heart or lung disease) [6]. As evidence shows, RSV is a serious healthcare challenge which up till now does not have an approved vaccine to efficaciously prevent infection. Presently, the only means of prevention are limited to environmental prophylaxis and a humanized monoclonal antibody administered to selected pediatric high risk patients.

Several studies have been carried out to understand the pathogenesis of the infection and the factors conditioning the severity of the disease, with the aim to improve prevention and treatment of the infection and to reduce RSV-related mortality and morbidity.

Clinical consequences of the infection are dependent on the virus, the environment, the host, and the immune response and as a result, interactions among these factors lead to different courses of the disease associated also with differences in inflammatory and immune response in the lung. The histological characteristics of the infection and the differences between nonsevere cases and lethal cases are well expressed in two studies reporting autopsy data [7, 8]. The first report documented an infant who died from an acute trauma after an acute RSV infection [7] and the other one was on 11 infants who died from a severe RSV infection [8]. In the first study, the infection was self-limiting, while in the second study the infection was extremely severe and led to the death of all 11 infected infants. In the severe cases, RSV antigen was found in exfoliated alveolar cells associated with high levels of neutrophils and monocytes and a low number of T cells, while in the nonfatal case, the antigen was found only in the bronchial epithelial cells and the infiltrate was composed predominantly by CD8+ T cells and B cells.

Several studies have analyzed the pathogenesis of RSV infection in animal models with the aim to confirm the hypotheses about the mechanisms of the infection and the onset of respiratory symptoms. Ideally, an animal model of the infection provides the means to study the entire spectrum of the disease, consisting of clinical features of illness with appropriate histopathological and physiological characteristics, including relevant pathogenic mechanisms. Taking into consideration that it is unlikely that a single animal model reproduces all these aspects of human disease, one needs to specify what features of human RSV disease are crucial and what are the advantages and the limits of animal models [9]. Studies performed on mice and cotton rats showed that infected mice developed mucus hyperproduction, increased airway hypersensitivity, airway remodeling, and Th2 cytokines and cellular responses [10]. Another study performed on neonatal mice showed that B- and T-cell mediated immune response was weaker in the neonatal period than in adult age and that RSV infection in the neonatal period causes a marked deviation of the subsequent T CD4+ cell response leading to the secretion of significant amounts of Th2 cytokines in addition to IFN [11], whereas study on mice demonstrated the immune-mediated pathogenesis of RSV infection. It was observed that T-cell depleted BALB/c mice had prolonged viral excretion after intranasal infection with human RSV, but the clinical course was less severe than the course observed in immunocompetent BALB/c mice [12]. There have been studies that showed the infection in lamb models, because of the possibility to obtain both preterm and term lambs and the similarity with the human respiratory system [13, 14]. The lungs of preterm lambs had an increased proinflammatory response after RSV infection, mediated by MCP-1, MIP-1*α*, IFN, TNF-*α*, and PD-L1 mRNA in comparison to the term lambs. Nitric oxide levels were lower in preterm than in full-term lambs, demonstrating alternative macrophage activation. Although infection induced a large neutrophil recruitment into the lungs of preterm lambs, neutrophils produced less myeloperoxidase than in full-term lambs, indicating decreased functional activation [15]. Studies conducted on animal models are of outstanding interest but each model poses limits because of the characteristic of animals, the availability of research tools, and the differences with humans. Clarke et al. in 1994 and Belshe et al. in 1977 [16, 17] studied RSV infection in a chimpanzee model. Given the genetic and anatomic similarity to humans this model proved to be quite significant, nevertheless limited by the economical, logistic, ethical, and emotional implication [9].

As previously reported, sheep and lambs have been used in several experimental settings [13, 15]: the size of the airways and the lungs and the development of the lungs in fetal and neonatal period are comparable to humans (2008), even if only few sophisticated molecular tools are available and the management of these animals requires large spaces and specific veterinary expertise. Cotton rats have also been largely used to study RSV infection and to develop specific neutralizing antibodies [18–20]. The disadvantages of cotton rats are the restricted pool of reagents and the low availability of transgenic or gene-deleted rats. Transgenic or gene deleted mice have been instead widely used in the previously cited studies [10–12]: the advantages of this model include the large experience and availability of sophisticated molecular tools. The limits of mice model are attributable to the molecular, genetic, and anatomic differences between mice and humans [21, 22]. Moreover, other studies that are comprised of two cognate host-pneumovirus models were analyzed: bovine RSV infection in cattle [23] and viral pneumonia in mice [24], which also served useful as they both have some similarities to human RSV infection.

Given that animal models are essential to understand the pathophysiology of RSV infection, it is clear that no animal model is ideal to completely clarify the mechanisms. For this reason studies performed on human fluids and tissues are essential to improve the knowledge of the infection. The following is a summary of the main findings pertaining to physiopathology of RSV infections in humans, particularly focusing on the lung response (Table 1).

## 2. Viral Factors 

RSV is an enveloped RNA virus of the Paramyxoviridae family with a single-stranded negative-sense RNA genome of 15.2 kb [25, 26]. There are five RSV proteins detectable in nucleocapsid structure and/or RNA synthesis: the nucleocapsid N protein that tightly encapsidates genomic RNA, the large L protein of the major polymerase subunit, the P phosphoprotein that is an essential cofactor in RNA, and the M2-1 and M2-2 proteins, respectively are involved in transcription and in modulating the balance between transcription and RNA replication [27]. Four other proteins are associated with the lipid bilayer: the matrix M protein which lines the inner envelope surface, the heavily glycosylated G, fusion F, and small hydrophobic SH proteins that are transmembrane surface glycoproteins. G and F are the only antigens involved in the virus neutralization [27, 28]. The G protein plays a key role in immune evasion of the virus. First, it is a highly glycosylated protein, which may interfere with immune recognition [28, 29]. It is also highly variable, which allows easy escape from neutralizing antibodies. Furthermore, a truncated, secreted form, sG, is produced during RSV infection and it binds RSV-specific antibodies reducing the concentrations available for RSV neutralization [30]. The G protein also has reduced homology to fractalkine and can reduce the action of host fractalkine and the influx of natural killer (NK) cells and CD4+ and CD8+ T cells [31]. The glycoprotein G mimics fractalkine and chemokine involved in the chemotaxis of leukocytes expressing its receptor [32]. For this reason some authors [32] hypothesized that RSV modulates effector functions of cytotoxic T cells and that disease severity is linked with CX(3)CR1 expression. Finally, the sG protein acts as a toll like, receptor (TLR) antagonist, resulting in the downregulation of their inflammatory response [33]. Several studies confirmed that G glycoprotein has important immune modulatory effects. For example, it has been shown that during RSV infection the G glycoprotein promotes a Th2 immune response in pulmonary CD3^+^ T cells (mediated by upregulation of IL-4 and IL-5) by negatively modulating Th1 cytokines (IFN-*γ* and IL-2) [31].

The virus has a single-serotype with two antigenic subgroups (A and B); the most divergent surface protein between subgroups A and B is G protein. The association between subgroup and severity of the disease is still controversial [28]. Both subgroups circulate during epidemic seasons with an alternate predominance every one or two years [28].

The tropism of the virus is extremely high for superficial cells of the respiratory tract. Pathological findings include necrosis of epithelial cells, proliferation of the bronchiolar epithelium, infiltrates of monocytes, T cells, and neutrophils between vessels and small airways. Syncytia, polynucleotide cells derived from the fusion of infected cells, are sometimes observed in the bronchiolar epithelium but are not common [7, 52]. NS2 protein was observed to be involved in the shedding of infected epithelial cells, resulting in an accelerated clearance of the virus from the mucosa, as well as in airway obstruction secondary to the accumulation of detached infected epithelial cells and increased mucous secretion [26]. Moreover, the NS1 and NS2 proteins downregulate the type 1-IFN response through the inactivation of the factor 3 [14].

## 3. Host and Environmental Factors 

The host response to RSV infections has largely been studied in infants with comorbidities (prematurity, immunodeficiency, congenital heart disease, and chronic lung disease) but not in healthy infants. In these populations, incomplete development and hyperreactivity of the airways, pulmonary congestion, and immunological impairments may play a role in the pathogenesis of a severe LRTI [14]. Moreover, several genetic polymorphisms of the genes involved in the immune response (innate defense, host cell receptors, neutrophil response, Th1/Th2 response, and adaptive immunity) have been identified as significant factors in the severity of RSV disease [53]. Other factors influence the severity of RSV disease in infants: young age at the onset of the RSV season (<3 months); tobacco smoke and pollution exposure; daycare attendance; presence of siblings; meteorological factors (temperature, humidity); and lack of breastfeeding [34–38, 54]. Recent studies suggest that low levels of vitamin D in cord blood of healthy neonates are associated with increased risk of severe RSV LRTI in the first year of life [39].

## 4. Immune Response 

Two different mechanisms are involved in the development of airway inflammation: first, airway inflammation was caused by the necrosis of airway epithelial cells subsequent to the cytopathological effect of RSV [41–44] and second, the immune response to RSV may directly damage the airways, resulting in inflammation and more airway destruction [44]. Innate immunity is firstly involved against virus infection, before induction of the adaptive immune response [45, 46]. It is well documented that innate immunity is critical to restrain virus spreading and infection. After RSV infection, the virus infects epithelial cells, alveolar macrophages, and dendritic cells, which trigger an innate antiviral response thought pattern recognition receptors including toll-like receptors (TLRs), nucleotide-biding oligomerization domain- (NOD-) like receptors (NLRs), and retinoic acid-inducible gene-I- (RIG-I-) like receptors (RLRs) [46]. This response induces the upregulation of cytokines and chemokines (IL-8, IP-10, MCP-1, MIP-1*α*, MIP-1*β*, RANTES, IL-6, TNF-*α*, IL-1*αβ*, and IFN-*αβ*) [14, 50] and surfactant and cell-surface proteins. This subsequently leads both to both a direct antiviral action and the activation of natural killer cells (NK), granulocytes, monocytes, macrophages, dendritic cells, and T lymphocytes. Neutrophils are the predominant cells found in the airways of RSV infected patients [44], since their prevalence in lavages taken during an episode of RSV bronchiolitis was observed to be 93% in the upper airways and 76% in the lower airways [55], respectively. Similar to other respiratory inflammatory diseases (such as asthma), neutrophils are recruited in the airways by chemokines, such as IL-8 that was found in high concentration in nasal lavages of infants during RSV bronchiolitis [56]. The neutrophils are responsible of the damage of the airways resulting in sloughing of the epithelium [57]. Eosinophils also play a role in the pathogenesis of the inflammatory response following a RSV infection. Elevated levels of eosinophilic cationic protein (ECP) in serum and nasal secretions have been found [58], even if an increase in eosinophils was not found in lavages. It is possible that eosinophils remain in the mucosa, while their secreted products are detectable in the secretions.

Besides the innate immune response previously described, the adaptive immune response is activated after RSV infection and it plays an important role in the clinical course of the infection and in the protection against subsequent reinfections [59]. The immune response induces the synthesis of secretory immunoglobulins A (IgA) that are protective for the upper respiratory tract and serum IgG antibodies that are more efficient in protecting the lower respiratory tract [47]. Secretory IgA play an important role in protecting the upper respiratory tract, where the access of serum IgG is difficult [48]. The IgA response is short-lived following primary infection, but it is longer after an episode of following reinfection [47]. Protective serum IgG antibodies determine a substantial but limited protection to RSV infection; the short duration of the protective antibody titre may explain the high incidence of reinfections after a first episode of natural infection. The production of protective IgG antibodies increases after reinfections, as demonstrated by the high antibody titers measured in adult populations [49]. In addition to producing antibody secretion, CD4+ and CD8+ T cells promote viral clearance mediated by IFN-*γ* secretion [25].

Dendritic cells (DC) also play an important role in the activation of the host adaptive immune response [60]: after RSV infection lung-resident DCs incorporate viral antigens, migrate to the lymph nodes draining from the lung, and activate the T-cell response. Poor T-cell responses were observed to play a key role in the pathogenesis of severe RSV infections [44] and lower T-cell counts in peripheral blood were observed to be positively correlated to a more severe course of RSV bronchiolitis [61]. Moreover, immune responses during RSV bronchiolitis requiring mechanical ventilation show low T-cell proliferative responses and IFN-*γ* secretion [62, 63]. The activation of CD8+ T cells is primarily mediated by dendritic cells [64]. In absence of inflammatory response, CD103+ and CD11b+ conventional DC and plasmacytoid DC are detectable in the lungs. In the inflammatory response, such as a respiratory viral infection, monocyte-derived DC are recruited to the lung and CD103+ DC migrate from the epithelium of the airways to the draining mediastinal lymph nodes to primarily induce the CD8+ T cell response against the invading virus [65, 66].

In a recent study, regulatory T cells (Tregs) were shown to be important in modulating the innate and adaptive responses during the later stages of RSV lung infection; depletion of Tregs before RSV infection determined delayed viral clearance and a severe clinical course in mice [14, 64, 67]. The secretion of interferon *αβ* (IFN*αβ*) along with the expression of the IFN*αβ* receptor (IFNAR) is crucial not only in limiting viral replication but also in activating the inflammatory response in the respiratory tract [68]. CD14 monocytes were also observed to play a key role in the control of antiviral type I IFN responses to RSV through a direct antibody mediated and an indirect infection mediated mechanisms [69]. With regard to CD4+ response, it was shown to be crucial for the course and the outcome of the disease, since the polarization of the CD4+ T cells towards a Th1 phenotype reduces viral replication and inflammatory response through the secretion of IFN-*γ* and IL-12, while the polarization towards a Th2 phenotype promotes the secretions of proinflammatory cytokines (IL-4, IL-5, and IL-13) and the recruitment of eosinophils, neutrophils, and monocyte [44]. These mechanisms demonstrate that the Th1/Th2 balance is crucial for the severity of the disease and the outcome and the host response is involved in the pathogenesis of the disease. The immune response itself, when characterized by a Th2 response, may contribute to the pathogenesis of severe infections. This may be explained through the analysis of pathogenetic mechanisms that occur in response to the administration of formalin-inactivated RSV vaccine (FI-RSV) and in RSV-infected immunocompromised hosts. In the 1960s, an experimental vaccine against RSV inactivated with formalin was administered to a cohort of 219 children including four-month-old infants and their siblings <10 years and to a group of healthy infants between the ages of 6 and 24 months [70]. In the following RSV epidemic season, 80% of the FI-RSV vaccinated children had a natural RSV infection and were hospitalized; two of them died [70–72]. Postmortem examination of the lungs showed an extensive mononuclear cell and eosinophils infiltrate. Moreover, a higher eosinophils number was found in the blood of vaccinated infants than in blood of control children [70]. The immunological explanation of this episode was subsequently formulated through a murine model [73]. The lack of protective effect of FI-RSV vaccine and its correlation to a more severe infection were probably due to several factors, including inadequate development of incomplete affinity maturation of the antibodies, poorly neutralizing antibodies against RSV encoded epitopes, and lack of an anti-RSV cytotoxic T lymphocyte response [73]. The response to FI-RSV vaccine was associated with the activation of IL-4 secreting CD4+ cells with subsequent Th2 response determining more significant pulmonary damage and a marked eosinophilic infiltration. This experience with FI-RSV vaccine demonstrated that an exaggerate Th2 response is related to the severity of the RSV infection since it determines exacerbate inflammatory response. Moreover, children with T-cell deficiencies have an impaired viral clearance, and therefore they are at higher risk for severe and potentially lethal viral infections, including RSV LRTI [74]. Several authors have reported cases of increased morbidity and mortality after an acute RSV infection in comparison to immunocompetent infants [40].

## 5. Nonimmunological Factors 

The anatomy of the airways predisposes young infants to severe RSV infections. The symptoms are subsequent to inflammatory response to RSV in the lung that causes the narrowing of the airways. Sloughed necrotic epithelium and excessive mucus secretion increase the airway obstruction and the formation of mucus plugs. This determines air trapping and hyperinflation or collapse of distal lung tissue [50]. It has been hypothesized that some infants have relatively smaller airways that make them predisposed to both RSV LRTI and recurrent lower respiratory symptoms in the first years of life [75]. Recently, RSV has been shown to determine increased sensitivity to the proinflammatory effects of substance P by upregulating neurokinin-1 gene receptor expression in several cells including endothelial cells, lymphocytes, macrophages, and mast cells [50]. Growing evidence has demonstrated a crucial role of the pulmonary surfactant in the pathophysiology of RSV LRTI [51]. In particular, the surfactant proteins SP-A and SP-D influence the mechanisms of lung innate immunity against RSV infection. Modifications in lipid components and hydrophobic proteins alter the surface tension at the alveoli and terminal bronchioles and determine an increase effort in breathing resulting in respiratory distress [51].

## 6. Conclusions

The pathogenesis of RSV disease is multifactorial and is influenced by viral factors, environmental factors, host factors, and genetic factors of the host that interact to determine the severity and the outcome of the illness. Risk factors for infection have been identified (exposure to epidemic season, crowding, presence of siblings, daycare attendance etc.) and associated with a severe course of disease (immunodeficiency, low gestational age, low birth weight, malformations, and young age at the beginning of the epidemic season). Although the pathogenetic mechanisms are not completely clear, they could be very useful in improving therapeutic strategies and provide means to come up with an effective and safe vaccine. Several animal models have provided findings that have proven to be useful in investigating the lung response to RSV infections; however, it is important that the limitations of each animal model should be taken into consideration and that the findings obtained in animal models may require, when possible, confirmation in studies using human fluids and tissues.

## Figures and Tables

**Table 1:** Factors involved in the lung response to respiratory syncytial virus infections.

Viral factors	Host and environmental factors	Immunological factors	Nonimmunological factors
(i) Subgroup [28] (ii) Viral load and high infectivity [4] (iii) Immune evasion mediated by G protein [28–32] (iv) NS1 and NS2 proteins [14]	(i) Preexisting diseases [34, 35] (ii) Young age [34, 35] (iii) Exposure to tobacco smoke and traffic-derived pollution [34, 35] (iv) Lack of breastfeeding [36] (v) Daycare attendance [34, 35] (vi) Siblings [34, 35] (vii) Meteorological factors [37, 38] (viii) Low levels of vitamin D [39] (ix) Immunodeficiency [40]	(i) Innate immunity (neutrophils, eosinophils, and complement) [41–46] (ii) Adaptive immunity (IgA, IgG, and T lymphocytes) [47–49] (iii) Th1/Th2 balance [44]	(i) Anatomy of the airways in young infants [50] (ii) Surfactant proteins [51]
